# Invasive Pulmonary Aspergillosis as a Consequence of Influenza A Infection and Refractory Septic Shock: A Case Report

**DOI:** 10.7759/cureus.104368

**Published:** 2026-02-27

**Authors:** Kristoffer Brustad, Francisco Adragao, Diogo Santos, David Nora

**Affiliations:** 1 Internal Medicine, Unidade Local de Saúde Lisboa Ocidental - Hospital de Egas Moniz, Lisbon, PRT; 2 Intensive Care Unit (UCI-4), Unidade Local de Saúde Lisboa Ocidental - Hospital de São Francisco Xavier, Lisbon, PRT

**Keywords:** cardiogenic shock, ecmo, glucocorticoids, intensive care, invasive mechanical ventilation, invasive pulmonary aspergillosis, refractory shock, septic shock, vasopressor

## Abstract

Glucocorticoids are commonly administered in refractory septic shock because of their potential hemodynamic benefits. However, their immunosuppressive effects carry a recognized risk of opportunistic infections. In the presence of concomitant viral infections, such as influenza A, which themselves induce local immune dysregulation, this risk may be amplified. The combined immunological impairment can create a permissive environment for opportunistic pathogens, such as aspergillosis, potentially worsening clinical outcomes. We present the case of a 59‑year‑old man with type 2 diabetes mellitus, admitted to the intensive care unit (ICU) for severe community‑acquired pneumonia, complicated by euglycemic diabetic ketoacidosis, septic shock evolving into mixed shock (septic and cardiogenic), and acute respiratory failure. He received broad‑spectrum antimicrobial therapy and required invasive organ support, including invasive mechanical ventilation upon admission. Due to refractory respiratory failure and shock, he was referred and transferred to a reference center for veno‑arterial‑venous extracorporeal membrane oxygenation, where he was given seven days of systemic glucocorticoid therapy. After initial clinical and hemodynamic improvement, the patient developed invasive pulmonary aspergillosis (IPA) with cavitation, confirmed by positive galactomannan (3.1) on bronchoalveolar lavage and characteristic findings on thoracic CT scan. He progressed to multiorgan failure and died 24 days after admission to the ICU. This case highlights the narrow therapeutic balance between the hemodynamic benefits of glucocorticoids in refractory septic shock and the increased risk of opportunistic infections. IPA is increasingly recognized in critically ill patients without classical immunosuppression, particularly in the setting of severe influenza and corticosteroid exposure. Given the modest mortality benefit and potential for serious infectious complications, glucocorticoid therapy should be carefully individualized, with further studies needed to refine patient selection and treatment strategies.

## Introduction

Refractory septic shock remains one of the leading causes of mortality in intensive care units (ICUs), despite advances in antimicrobial therapy, hemodynamic support, and advanced organ‑support technologies [1]. The use of systemic glucocorticoids has been widely discussed in the cases of refractory septic shock requiring persistent vasopressor support [1]. However, there is minimal evidence of a true impact on reducing mortality [2], and the risks associated with their use, particularly the increased susceptibility to opportunistic infections, are well established [3].

In recent years, the epidemiological profile of invasive fungal infections has changed markedly, with a rising proportion of invasive pulmonary aspergillosis (IPA) cases reported in critically ill patients admitted to ICUs, especially in the context of corticosteroid use and viral respiratory infections, such as influenza [3].

We present a clinical case aimed at discussing the role of systemic corticosteroid therapy in septic shock, in light of the increased risk of invasive fungal infections, particularly IPA. 

## Case presentation

We present the case of a 59‑year‑old man with a history of insulin‑treated type 2 diabetes mellitus, treated and compliant with empagliflozin, without target‑organ complications, benign prostatic hyperplasia, and beta-lactam allergy, who presented to the emergency department with a fever of 39ºC, productive cough, and progressive dyspnoea in the last four days. Physical examination revealed a blood pressure (BP) of 110/70 mmHg, tachypnea with a respiratory rate (RR) of 25 cpm, hypoxemia of 88% with 2 L/min of supplemental oxygen on nasal canula, and pulmonary auscultation with coarse breath sounds, rhonchi, and bilateral crackles. No additional symptoms or signs were reported.

Upon admission, complementary diagnostic tests were performed, as presented in Table 1, revealing leukopenia with neutropenia and an elevated C‑reactive protein (CRP). Arterial blood gas analysis under oxygen therapy (6 L/min), as presented in Table 2, showed a primary metabolic acidosis with respiratory compensation and hypoxia, hyperlactatemia, and hyperglycemia in a patient with elevated ketonemia (7.4 mmol/L (reference value: 0-0.5 mmol/L)). Chest radiography, CT, and CT angiography of the thorax demonstrated bilateral diffuse peribronchial consolidation predominantly affecting the lower lobes, along with mediastinal lymphadenopathy (the largest measuring 11 mm).

**Table 1:** Blood work results at admission

Parameter	Value at admission	Reference value
Leukocytes	1.3 x 10^9 ^cells/L	4.0-10.0x10^9^ cells/L
Neutrophils	0.9 x 10^9^ cells/L	1.5-7.0x10^9^ cells/L
C-reactive protein	10.4 mg/dL	<0.5 mg/dL
Creatinine	1.04 mg/dL	0.70-1.20 mg/dL

**Table 2:** Arterial blood gas on admission with 6 L/min of supplemental O2

Parameter	Value at admission	Reference value
pH	7.32	7.35-7.45
PaCO2	27 mmHg	35-45 mmHg
PaO2	69 mmHg	70-100 mmHg
HCO3 std	13 mmol/L	21-28 mmol/L
Glycemia	209 mg/dL	60-180 mg/dL
Lactate	2.2 mmol/L	0.5-2.0 mmol/L
Anion gap	15.9 mmol/L	7-16 mmol/L

A diagnosis of sepsis secondary to severe community‑acquired pneumonia with type I respiratory failure, complicated by euglycemic diabetic ketoacidosis, was made. Sputum and blood cultures were collected, and fluid resuscitation, empirical intravenous (IV) antimicrobial therapy with levofloxacin 750 mg daily, and insulin infusion were initiated.

The patient deteriorated with worsening hypoxemia and respiratory fatigue, requiring orotracheal intubation and invasive mechanical ventilation. Subsequently, he was admitted to the ICU.

Upon ICU admission, he demonstrated further clinical deterioration, progressing to septic shock with vasopressor requirement (norepinephrine 10 µg/min) and escalating FiO_2_ up to 90%. Ventilatory mechanics showed: static compliance 35 mL/cmH2O, time constant 0.95 seconds, and driving pressure of 13. Sedation and analgesia were increased, neuromuscular blockade was initiated, and prone positioning was applied.

A Polymerase Chain Reaction (PCR) respiratory viral panel and atypical pathogen screen was performed, yielding a positive result for Influenza A. Oral Oseltamivir 75mg twice per day was added to the antimicrobial regimen and maintained for 10 days. A tracheal aspirate sample was also obtained, growing methicillin‑sensitive Staphylococcus aureus.

Despite these interventions, further clinical deterioration occurred with progressive shock requiring increased vasopressor support (norepinephrine 50 µg/min). A PiCCO catheter was inserted for hemodynamic assessment, revealing serum lactate of 3.2 mmol/L, central venous saturation of O_2_ (ScvO_2_) 70%, CO_2_ gap of 11 mmHg, prolonged capillary refill time, cardiac index of 2.4 L/min/m², Global End Diastolic Index (GEDI) of 688 mL/m², systemic vascular resistance index (SVRi) of 1300 dyn·s·cm⁻⁵·m², and extravascular lung water index (ELWI) of 12 mL/kg. Echocardiography showed severely depressed ejection fraction (20%) and reduced cardiac output (velocity-time integral (VTI) = 9 cm).

Given the worsening mixed‑type shock (septic and cardiogenic), argipressin was added for vasopressor support.

Following microbiological confirmation from the tracheal aspirate and in view of the worsening shock, Panton‑Valentine leukocidin (PVL) testing was requested, and antibiotic therapy was escalated to IV linezolid 600 mg twice per day after 48 hours of levofloxacin, and maintained for 12 days. Due to refractory metabolic acidosis with renal impairment, continuous venovenous haemodialysis (CVVHD) was initiated, using regional citrate anticoagulation with calcium supplementation.

Hemodynamic instability persisted, accompanied by electrical instability resulting in supraventricular tachycardia. Attempts at cardioversion led to degeneration into pulseless ventricular tachycardia. Advanced life support was initiated, with ROSC achieved after three defibrillation cycles, followed by atrial fibrillation with rapid ventricular response. A second electrical cardioversion restored sinus rhythm, without improvement in blood pressure profile.

Due to refractory hypoxemia (PaO₂/FiO₂ 160) despite 16 hours of prone positioning, hemodynamic and electrical instability, the patient was accepted for transfer to a specialized ECMO center and started on veno‑arterial‑venous extracorporeal membrane oxygenation (V‑A‑V ECMO), 74 hours after admission to our ICU.

During his ECMO admission, he progressed to necrotizing pneumonia, with positive PVL results. IV Clindamycin 600 mg three times per day was added to antimicrobial therapy and maintained for 11 days. He remained in mixed shock requiring escalating doses of vasopressors (norepinephrine, argipressin, and methylene blue), fluid resuscitation with albumin, and initiation of glucocorticoids (hydrocortisone 50 mg every six hours), which were maintained for a total of seven days. Complications included well‑demarcated, irreversible ischemia of the lower limbs (without indication for urgent surgery), and pancytopenia (hemoglobin, leukocytes, and platelets).

Gradually, he experienced recovery of cardiac function with hemodynamic and respiratory improvement, resulting in a marked reduction in vasopressor requirements. However, ventilator weaning was prolonged and difficult due to severe critical‑illness myopathy, accompanied by hyperactive delirium.

**Figure 1:**
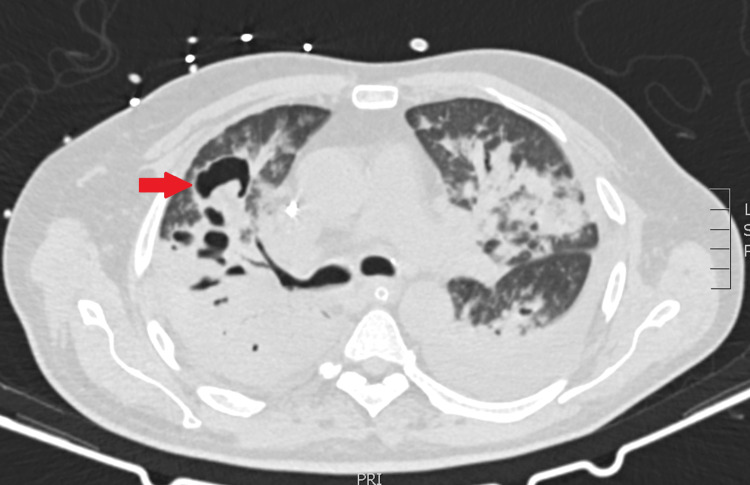
Horizontal section of the lung, involving multiple cavitary lesions, including one with the presence of an aspergiloma

**Figure 2:**
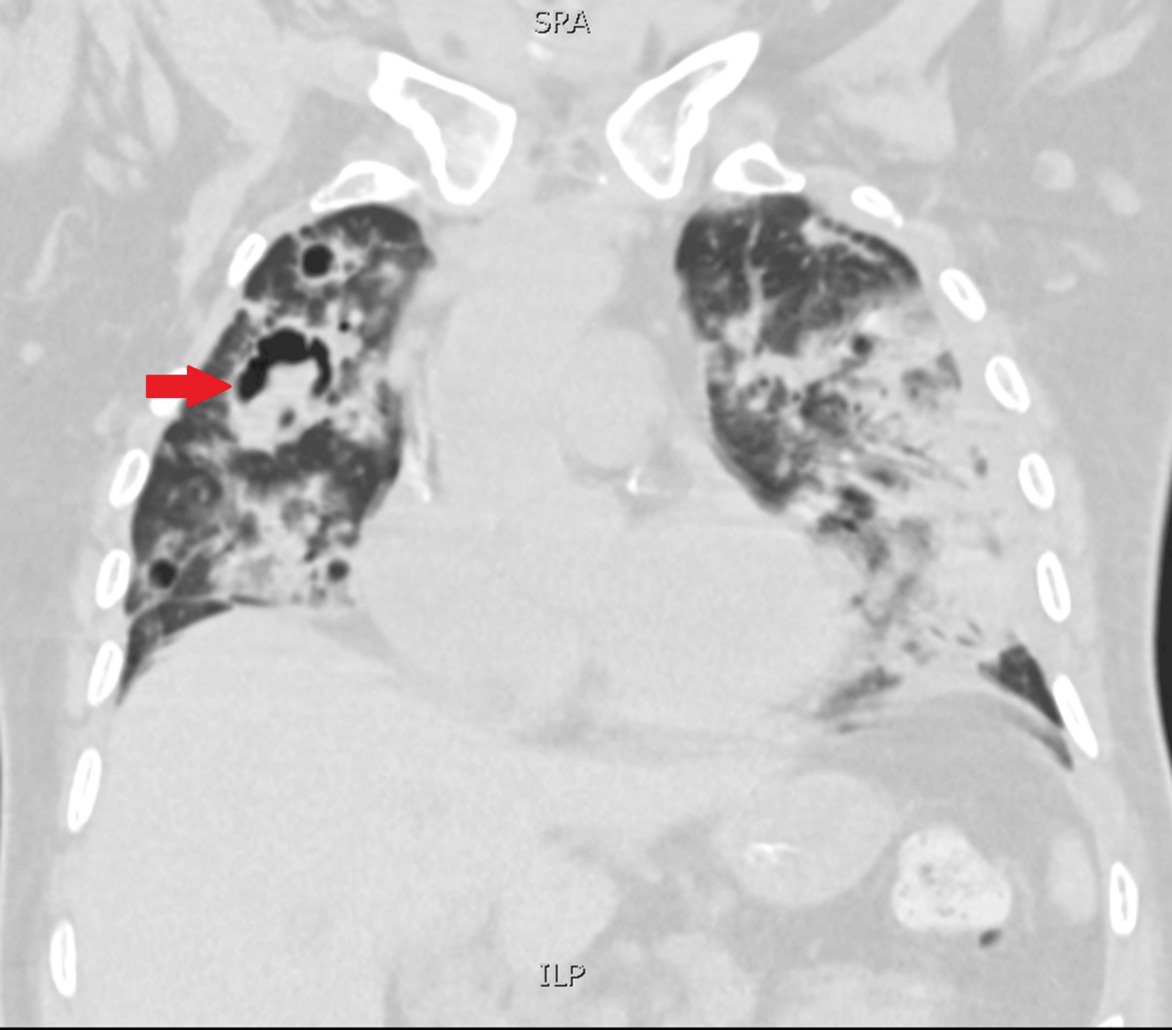
Coronal section of the lung with the presence of multiple cavitary lesions, including one with the presence of an aspergiloma

He was extubated after returning to the referring ICU, showing initial clinical improvement. However, he later developed fever, rising inflammatory markers, and production of abundant purulent respiratory secretions with ineffective cough, prompting re‑intubation five days after extubation. Repeat respiratory cultures by bronchoalveolar lavage (BAL) revealed multisensitive *E. coli *and positive galactomannan with a value of 3.1 (reference range <1). Chest CT identified IPA with cavitary lesions (Figures 1, 2). Targeted therapy with IV levofloxacin 750 mg per day and IV voriconazole 300 mg twice per day was initiated and maintained until death (four days).

The clinical course was unfavorable, with progression to multiorgan failure, a severe restrictive respiratory pattern and refractory hypercapnia and hypoxemia. Radiological deterioration showed extensive bilateral pulmonary consolidation with multiple cavities. The patient ultimately died, 24 days after admission in the ICU.

## Discussion

This case represents a common reality in intensive care medicine. Despite all available efforts and resources, it was not possible to save the patient’s life. Therefore, we consider it essential to critically analyze all aspects of this case, with the aim of identifying and correcting any potential failures or inconsistencies that may have occurred.

According to current recommendations outlined by the Surviving Sepsis Campaign (2021), hydrocortisone use is suggested in adults with refractory septic shock and ongoing vasopressor requirements, despite adequate fluid resuscitation. This recommendation is based on studies demonstrating faster shock resolution and an increased number of vasopressor‑free days. However, to date, no study has definitely shown a benefit in short‑ or long‑term mortality. The optimal agent, dose, and duration of corticosteroid therapy also remain undefined. A dose of hydrocortisone 200 mg/day is suggested, although the ideal duration is unclear. Therefore, glucocorticoid use in refractory septic shock remains a weak recommendation supported by moderate‑quality evidence [1].

A 2024 focused guideline update on corticosteroid use in septic shock reported that glucocorticoid therapy may be associated with modest reductions in both short-term mortality (≤30 days or in-hospital mortality; RR 0.93, 95% CI 0.88-0.99) and long-term mortality (>60 days; RR 0.94, 95% CI 0.89-1.00), based on pooled data from 46 randomized controlled trials. Notably, no credible subgroup differences were identified according to corticosteroid type, dose, or duration. However, the overall effect size is small, and high-dose short-course regimens (>400 mg/day hydrocortisone-equivalent for <3 days) are not recommended. These findings support the use of low- to moderate-dose corticosteroids in refractory septic shock, while underscoring the need to balance potential haemodynamic benefits against the risk of secondary infections [4].

According to a narrative review published in 2025, entitled "The Current Standard of Care for Septic Shock," a mortality benefit at 90 days was observed in patients treated with hydrocortisone 200 mg/day combined with fludrocortisone 50 µg/day. However, fludrocortisone monotherapy did not demonstrate any mortality benefit. Importantly, the article highlights the heterogeneity of the study population, showing a more favorable response in patients with septic shock secondary to community‑acquired pneumonia, whereas those with abdominal or urinary sources did not show significant improvement. Given the lack of robust evidence, this combination remains outside official guidelines and requires further research [2].

Glucocorticoids are a recognized risk factor for fungal infections, including cryptococcosis and other invasive fungal infections, due to their immunosuppressive effects [5-6]. A systematic review and meta‑analysis published in 2023 showed worse clinical outcomes in invasive aspergillosis (IA) in patients receiving supraphysiologic doses of glucocorticoids. A sub analysis of the same study also indicated that doses greater than 2 mg/kg/day of prednisolone‑equivalent, significantly increased mortality risk [7].

In recent years, IPA has ceased to be a condition exclusive to patients with classical immunosuppression, such as profound neutropenia, hematologic malignancies, or transplant recipients, and is increasingly recognized in ICU populations without conventional risk factors. Contributing factors include the growing use of corticosteroids and other immunomodulators, the physiological stress of critical illness (especially in mechanically ventilated patients), the prevalence of chronic lung disease, severe viral infections (e.g., SARS‑CoV‑2 or influenza), and metabolic comorbidities such as diabetes [3-8].

Among critically ill patients, systemic corticosteroid exposure is one of the strongest risk factors for IPA. In ICU populations, corticosteroid use for underlying conditions such as acute COPD exacerbations or septic shock is strongly associated with the development of IPA. In addition, systematic reviews suggest that even corticosteroid therapy administered during hospitalization carries increased IPA risk, particularly when treatment is prolonged or involves high doses [3-9].

The impact of glucocorticoids on IPA risk extends beyond generalized immunosuppression. These agents profoundly alter antifungal immune responses by suppressing critical signalling pathways such as NF‑κB and reducing TNF‑α production, mechanisms essential for effective neutrophil and macrophage recruitment. Simultaneously, they promote a Th2‑polarized immune profile via increased IL‑10 production, further weakening host defences against *Aspergillus*. This dual effect contributes to the susceptibility to fungal tissue invasion even in patients not traditionally considered immunocompromised [3].

Influenza has emerged as an important predisposing factor for IPA, particularly in critically ill patients. In a meta-analysis including 6,024 hospitalized influenza patients, the pooled incidence of IPA was approximately 10%, increasing to 11% among those requiring ICU admission. Importantly, influenza-associated IPA was associated with a pooled mortality of 52%, and patients who developed IPA had a 2.4-fold higher risk of death compared with those without fungal superinfection. Identified risk factors included influenza A (especially H1N1), chronic lung disease, smoking history, and prior corticosteroid exposure. In the context of severe influenza and concomitant immunomodulatory therapy, these findings underscore the substantial risk of secondary fungal infection and highlight the need for vigilance when corticosteroids are administered in critically ill patients [10].

In the patient described in this case, several of these risk factors were present, such as initial Influenza infection (causing respiratory epithelial damage and impaired local immunity), invasive mechanical ventilation (associated with significant physiological stress), and corticosteroid therapy. These factors act synergistically, creating the ideal environment for IPA development.

Furthermore, the diagnosis of IPA in critically ill patients remains challenging due to a heterogeneous clinical presentation and the absence of “easy” early diagnostic tools. A combined diagnostic strategy is often required, including microbiological analysis of respiratory secretions and galactomannan testing in bronchoalveolar lavage fluid. Early imaging findings on plain radiography or CT are often nonspecific, with more characteristic findings (halo sign, air‑crescent sign, or cavitation) appearing later in the disease course. Mortality associated with IPA in ICU settings is high and, in some studies, exceeds that observed in classical neutropenic populations [3].

## Conclusions

Given the available body of evidence, despite the potential benefits of reducing vasopressor requirements in refractory septic shock, the use of glucocorticoids must be carefully weighed. In the absence of consistent evidence demonstrating a mortality benefit, this case report highlights the risks associated with this therapy and its potential negative impact on clinical outcomes. This case questions current guideline recommendations and reinforces the need for further studies evaluating the effect of corticosteroid therapy on morbidity and mortality in patients with refractory septic shock.
